# Neuromedin U-deficient rats do not lose body weight or food intake

**DOI:** 10.1038/s41598-022-21764-6

**Published:** 2022-10-27

**Authors:** Kyoka Yokogi, Yuki Goto, Mai Otsuka, Fumiya Ojima, Tomoe Kobayashi, Yukina Tsuchiba, Yu Takeuchi, Masumi Namba, Mayumi Kohno, Minami Tetsuka, Sakae Takeuchi, Makoto Matsuyama, Sayaka Aizawa

**Affiliations:** 1 Department of Biology, Graduate School of Natural Science and Technology, Okayama University, 3-1-1 Tsushimanaka, Kitaku, Okayama 700-8530 Japan; 2 Department of Natural Sciences and Biology, Kawasaki Medical School, 577 Matsushima, Kurashiki, Okayama 701-0192 Japan; 3 Division of Molecular Genetics, Shigei Medical Research Institute, 2117 Yamada, Minami-ku, Okayama 701-0202 Japan

**Keywords:** Endocrine system and metabolic diseases, Feeding behaviour, Circadian rhythms, Molecular biology

## Abstract

Studies in genetically modified mice establish that essential roles of endogenous neuromedin U (NMU) are anorexigenic function and metabolic regulation, indicating that NMU is expected to be a potential target for anti-obesity agents. However, in central administration experiments in rats, inconsistent results have been obtained, and the essential role of NMU energy metabolism in rats remain unclear. This study aims to elucidate the role of endogenous NMU in rats. We generated NMU knockout (KO) rats that unexpectedly showed no difference in body weight, adiposity, circulating metabolic markers, body temperature, locomotor activity, and food consumption in both normal and high fat chow feeding. Furthermore, unlike reported in mice, expressions of *Nmu* and NMU receptor type 2 (*Nmur2*) mRNA were hardly detectable in the rat hypothalamic nuclei regulating feeding and energy metabolism, including the arcuate nucleus and paraventricular nucleus, while *Nmu* was expressed in pars tuberalis and *Nmur2* was expressed in the ependymal cell layer of the third ventricle. These results indicate that the species–specific expression pattern of *Nmu* and *Nmur2* may allow NMU to have distinct functions across species, and that endogenous NMU does not function as an anorexigenic hormone in rats.

## Introduction

Excessive energy intake is a cause of obesity. Feeding and energy metabolism are complexly regulated by various factors, including hypothalamic anorexigenic and orexigenic neuropeptides. One of these neuropeptides, Neuromedin U (NMU), is an anorexigenic factor and is expected to be a potential target for anti-obesity agents^1,2^. NMU was first identified in the porcine spinal cord by the uterine smooth muscle contractile activity assay in 1985^3^. The mature form of NMU consists of 25 amino acid residues in humans and 23 amino acid residues in mice and rats, each containing the highly conserved core active C-terminal domain of eight amino acid residues^4–6^. This domain is highly conserved among various species of mammals, birds, amphibians, and fish, suggesting that NMU plays an important role in endocrine functions^5–8^.

NMU is highly expressed in the gastrointestinal tract, brain, and pituitary gland^9–12^, and moderately expressed in the testis, ovary, thyroid gland, spleen, lymphocytes, adipose tissue, endothelial cells, and pancreas^12,13^. The wide distribution of expression suggests that NMU has multiple physiological functions in addition to smooth muscle contraction.

A series of subsequent studies has revealed many physiological roles of NMU, including feeding and energy expenditure^2^, stress responses^14^, circadian rhythmicity^15^, tumorigenesis^16^, and inflammation^17,18^. In particular, many reports have shown that NMU plays an essential role in metabolic homeostasis, such as regulating body weight, feeding behavior, activity, and energy metabolism, which are closely related to the pathophysiology of obesity.

The role of NMU in regulation of energy homeostasis in mammals has been well established by both pharmacological and genetic analyses in mice. Intracerebroventricular (ICV) administration of NMU in mice suppresses dark-phase food intake and fasting-induced feeding^19^. Furthermore, to investigate essential roles of endogenous NMU by genetic analyses, NMU-deficient mice, which developed hyperphagia and obesity, were generated^20^. Additionally, transgenic mice overexpressing NMU are hypophagic and leaner than wildtype (WT) mice^21^. The exact physiological functions of NMU are yet to be clearly defined, but the phenotypes of genetically modified mice strongly support a role of NMU in energy balance.

Pharmacological studies in other vertebrates have shown that NMU inhibits food intake and increases energy expenditure. ICV administration of NMU in rats reduces food intake and body weight, but increases body temperature, motor activity, and heat production^22–24^. Moreover, acute central administration of NMU reduces food intake in rats^22,23,25^, chickens^26^, and quails^27^.

In contrast to these reports, inconsistent results have been obtained from administration experiments, especially in rats. ICV injection of NMU into 24 h-fasted rats decreases subsequent food intake and body weight gain, but not in free-feeding rats^28^. One study has also reported that chronic injection of NMU into the paraventricular nucleus (PVN) of free feeding rats does not influence food intake or body weight^29^. Taken together, the anorexigenic effect of central NMU administration may be a pharmacological effect that is exerted only temporarily under certain conditions.

We previously reported circadian rhythmic expression of *Nmu* mRNA in the rat pars tuberalis (PT) with higher expression levels during the light phase and lower levels during the dark phase due to melatonin-induced suppression^30^. Although further investigations are needed to prove our hypothesis, we suggest that NMU in PT is related to the feeding rhythm such as rats eating food mostly during the night period.

In this study, to investigate the role of endogenous NMU in rats, especially its functions related to energy homeostasis and food intake, including the circadian pattern, we generated *Nmu* knockout (KO) rats using the clustered regularly interspaced short palindromic repeats (CRISPR)/Cas9 system and genome editing via the oviductal nucleic acid delivery (GONAD) method. The resulting NMU KO rats unexpectedly showed no hyperphagia, obesity, or impaired circadian feeding patterns compared with WT littermates. Furthermore, expression of *Nmu* and its receptor, Neuromedin U receptor 2 (*Nmur2*), was hardly detectable in hypothalamic regions, which are involved in the regulation of feeding and energy metabolism. Our data indicate that NMU is not critically required for appetite or fat deposition and is likely not a central regulator of food intake in rats.

## Results

### Generation of NMU-deficient rats

**Figure 1:**
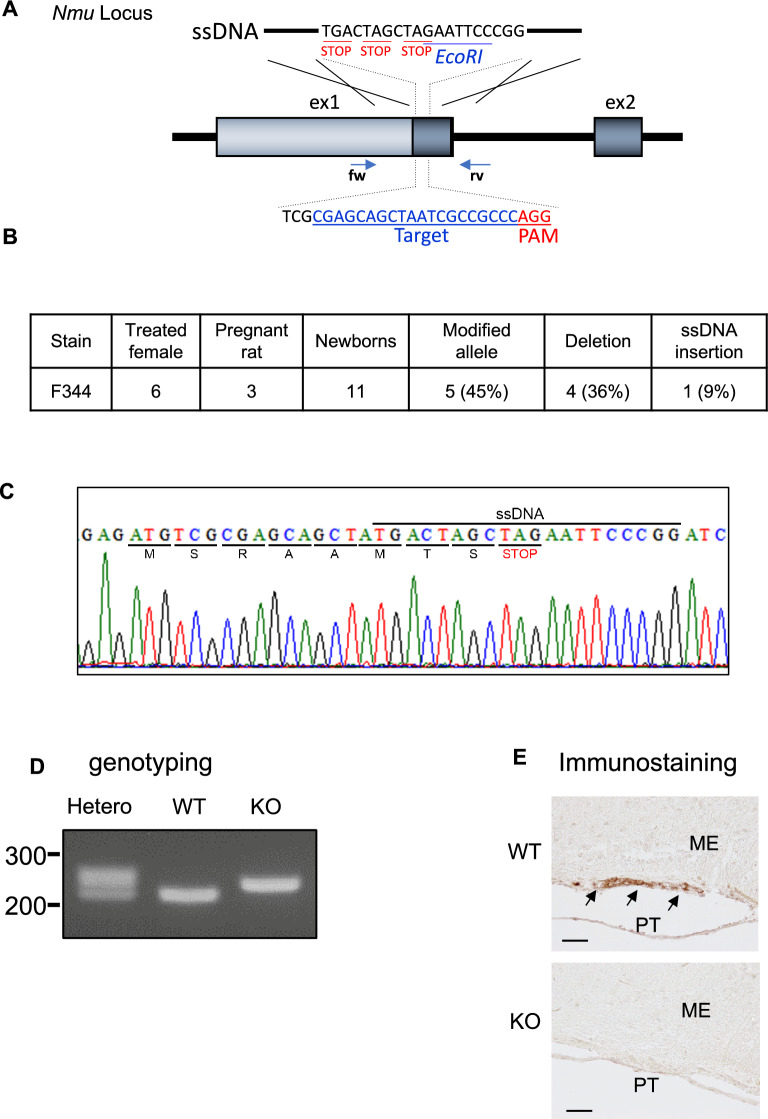
Generation of NMU-deficient rats. (**A**) Schematic diagram of the target sequence, PAM, and ssDNA at the *Nmu* gene locus. (**B**) Genome sequence of the mutation in the *Nmu* mutant allele. (**C**) Efficiencies of rat *Nmu* gene editing by the rGONAD method. (**D**) Genotyping of Hetero, WT, and KO performed by PCR. A representative gel image of genomic PCR products amplified from the indicated genotypes is shown. (**E**) Immunostaining of NMU in WT and NMU KO rat. The light micrograph is representative of IHC staining with the anti-NMU antibody in a frontal section of the brain. Positive signals are indicated by arrows. Scale bar: 50 µm. PT, pars tuberalis; ME, median eminence. See also Supplementary Figure S1.

NMU KO rats were generated by the rGONAD method as described previously^31,32^. Sequence analysis of the founder rat genome revealed that tandem stop codons were integrated 27 bases downstream of the first ATG (Fig. 1A). The mutation was verified by PCR followed by DNA sequencing, resulting in expression of only nine amino acids of NMU at its N-terminus (Fig. 1B). The rGONAD method was performed on six female rats and we successfully obtained one offspring with the ssODN insertion allele as a founder rat (Fig. 1C). WT and NMU KO rats used in this study were generated by mating *Nmu*^+/-^ heterozygous rats. Rats of each genotype were produced in accordance with Mendelian genetics. The genotype was confirmed by PCR (Fig. 1D). NMU immunohistochemistry was also performed on rat brain sections and intense signals were detected in WT rat PT (Fig. 1E, arrows), but not in NMU KO rat PT (Fig. 1E). These data indicated that the *Nmu* gene was functionally deleted.

### Effects of NMU deficiency on body weight, adiposity, and blood metabolic markers in normal chow-fed rats

**Figure 2:**
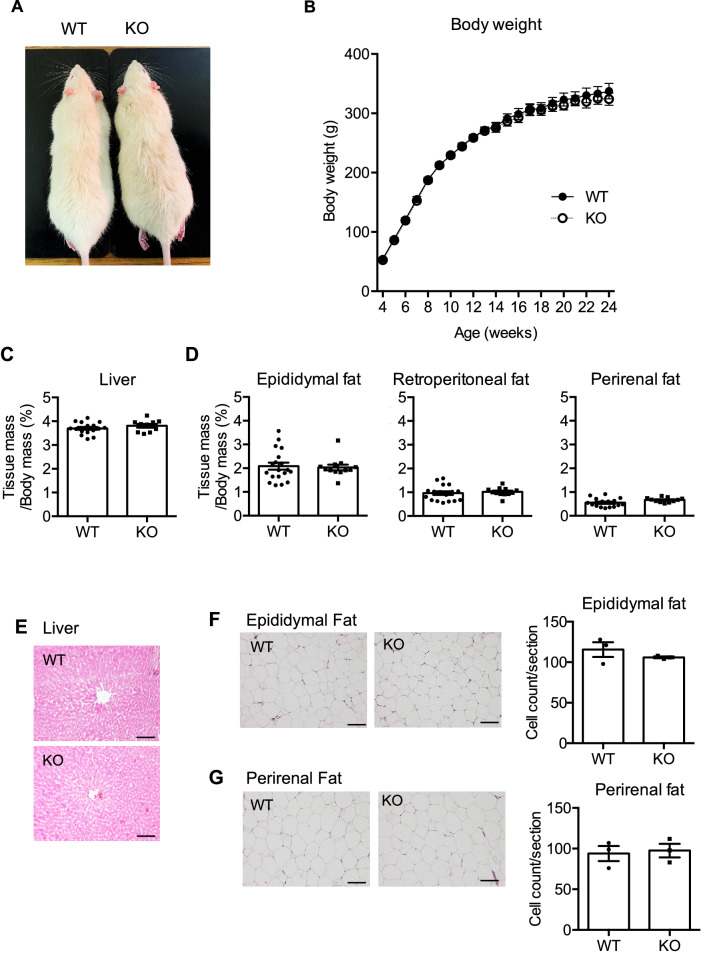
Body weight and adipose tissue mass of male WT and NMU KO rats fed normal chow. (**A**) Representative appearance of WT and KO rats (littermates) at 16 weeks of age. (**B**) Growth curve of WT and KO rats from 4 to 24 weeks of age (n = 15). (**C**) Percentage of the liver mass content in WT and KO rats (WT n = 16 KO n = 10). (**D**) Percentage of the visceral fat mass content in WT and KO rats (WT n = 19 KO n = 10). (**E**) Representative photographs of HE-stained liver sections of WT and KO rats. Scale bar: 100 µm. (**F**) Representative photographs of HE-stained sections of epididymal adipose tissue and the cell count per section (n = 3). Scale bar: 100 µm. (**G**) Representative photographs of HE-stained sections of perirenal adipose tissue and the cell count per section (n = 3). Scale bar: 100 µm. All data represent means ± SEM.

**Figure 3:**
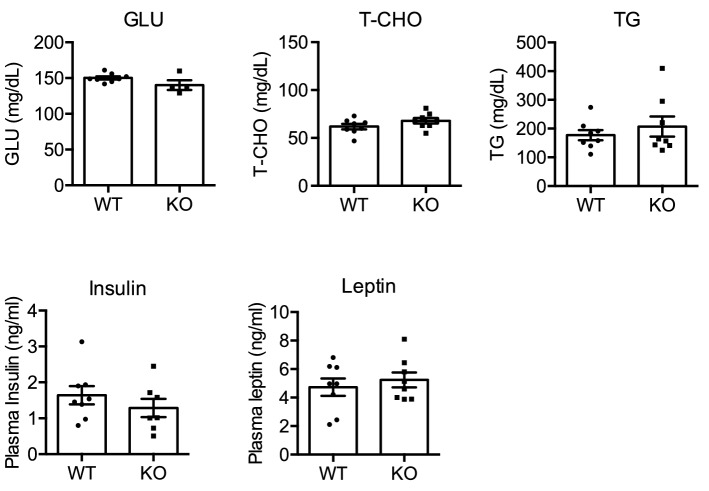
Blood biochemistry of male WT and NMU KO rats fed normal chow. Serum levels of GLU, T-CHO, and TGs were measured by an automatic biochemistry analyzer (n = 8). Plasma levels of insulin and leptin were measured by ELISAs (n = 8). All data represent means ± SEM.

NMU has been reported to be involved in energy metabolism, and therefore we first analyzed the physical characteristics of NMU KO rats. There were no deformities in NMU KO rats compared with WT littermates and their body length was normal (Fig. 2A). Unexpectedly, body weight gain, hyperplasia, or obesity was not observed in male NMU KO rats from 4 to 24 weeks of age (Fig. 2B). Female NMU KO rats also did not show the difference in the body weight gain (Supplementary Figure S3). Further analysis of adiposity showed no significant differences in the liver mass (Fig. 2C) or visceral fat mass (Fig. 2D). Histological analysis of the liver in NMU KO rats did not show any abnormalities, such as steatosis, which contains of large or many lipid droplets (Fig. 2E). Histological analysis of epididymal and perirenal fat showed that the adipocyte size in NMU KO rats was not different from that in WT rats (Fig. 2F,G). Because some reports show that blood test results are affected without a change in body weight, we next analyzed the levels of circulating blood metabolism markers. Unexpectedly, plasma levels of glucose (GLU), total cholesterol (T-CHO), and triglycerides (TGs) did not differ between WT and NMU KO rats (Fig. 3). Furthermore, circulating hormone levels, namely insulin and leptin levels, were not different between WT and NMU KO rats (Fig. 3).

### Effects of NMU deficiency on food consumption and the circadian feeding pattern

**Figure 4:**
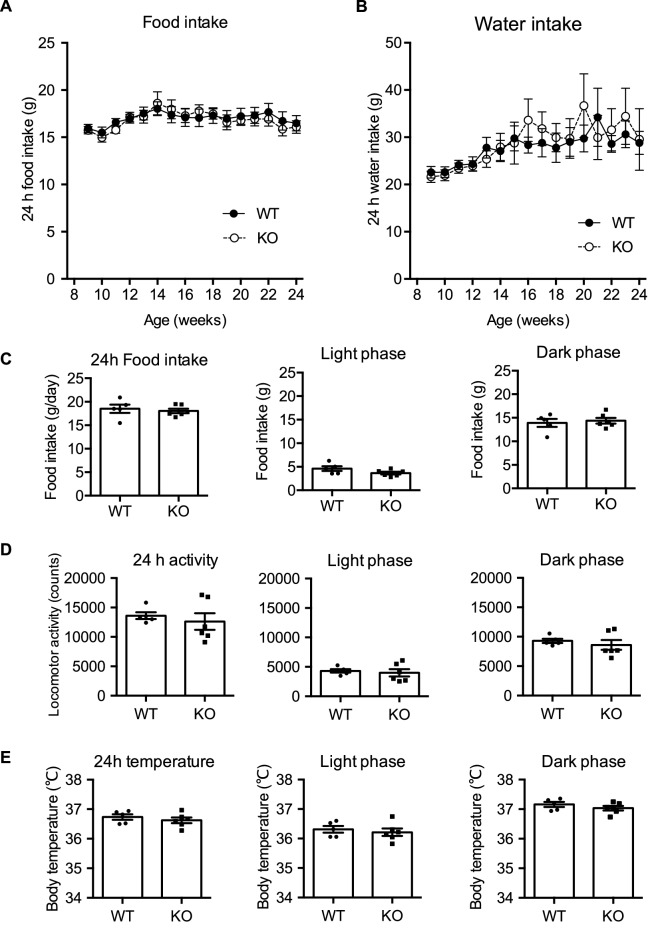
Food consumption, water intake, locomotor activity, and body temperature of male WT and NMU KO rats fed normal chow. (**A**) Food intake of free-feeding rats from 9 to 24 weeks of age (WT n = 22, KO n = 19). (**B**) Amount of water intake of free-feeding rats from 9 to 24 weeks of age (WT n = 22, KO n = 19). (**C**) Daily food consumption and feeding pattern at 14 weeks of age (WT n = 4, KO n = 5). (**D**) Counts of daily locomotor activity and activity pattern in the home cage (n = 5). (**E**) Mean body temperature over 24 h in light and dark phases (n = 5). All data represent means ± SEM.

NMU is an anorexigenic factor, and therefore we next analyzed the effect of NMU deficiency on food consumption and the feeding pattern with normal chow and water provided ad libitum. Unexpectedly, the daily food consumption of male NMU KO rats was not different from that of male WT rats at any measured age from 9 to 24 weeks (Fig. 4A). Female NMU KO rats also did not show the difference in the food consumption (Supplementary Figure S3). Additionally, the amount of daily water intake did not differ between WT and NMU KO rats (Fig. 4B). Next, we analyzed the daily feeding pattern because we had previously found a circadian rhythm of *Nmu* expression in the rat PT and considered that NMU may be related to the feeding rhythm^30^. Unexpectedly, no difference in food intake was found between KO and WT littermate rats in both light and dark phases (Fig. 4C). The light/dark ratio showed that not only WT rats, but also KO rats showed a clear circadian rhythm of feeding patterns [light/dark ratio (%): WT rats, 33.90 ± 4.39; NMU KO rats, 25.97 ± 2.78].

### Home cage locomotor activity and body temperature

Many previous reports have also shown that NMU plays a role in the regulation of energy metabolism. Typical home cage activity was measured by a telemetry system to determine whether NMU was associated with motor activity. The total 24 h-count of typical home cage activity in NMU KO rats was not different from that in WT rats. Cage activity was analyzed separately in light and dark phases, but no significant difference was observed. The average daily body temperature of NMU KO rats was not different from that of WT rats (Fig. 4D). Diurnal changes in body temperature, which is lower in the light phase than dark phase, were observed in both WT and NMU KO rats (Fig. 4E).

### Effects of NMU deficiency on body weight, energy metabolism, and feeding in high fat chow-fed rats

**Figure 5:**
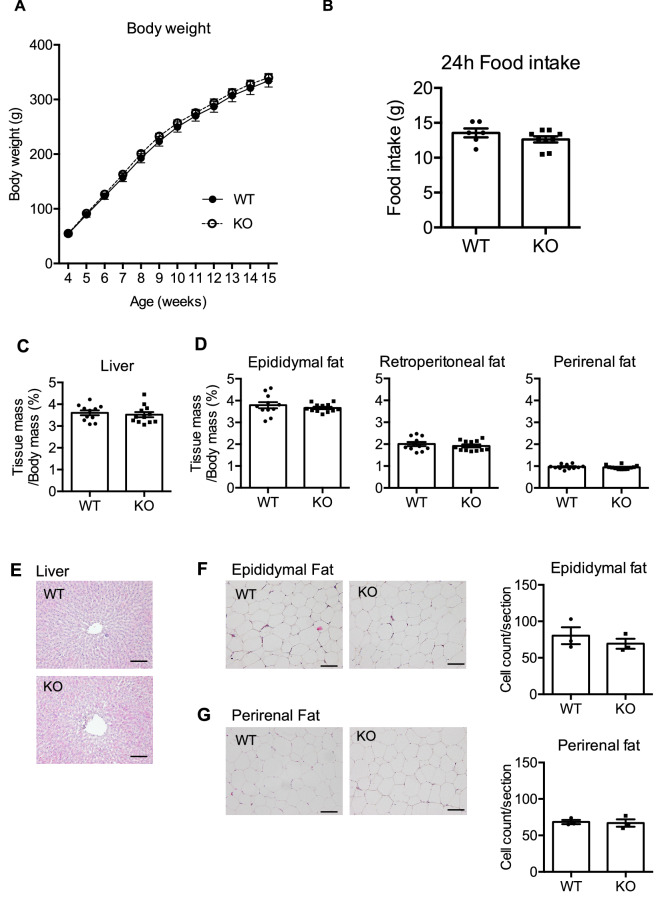
Body weights of male WT and NMU KO rats fed a high fat diet. (**A**) Growth curve of WT rats (n = 11) and KO rats (n = 12) from 4 to 15 weeks of age. (**B**) Food intake of free-feeding rats at 15 weeks of age (WT n = 22, KO n = 19). (**C**) Percentage of the liver mass content in WT and KO rats (WT n = 11 KO n = 12). (**D**) Percentage of the visceral fat mass content in WT and KO rats (WT n = 11 KO n = 12). (**E**) Representative photographs of HE-stained liver sections of WT and KO rats. Scale bar: 100 µm. (**F**) Representative photographs of HE-stained sections of epididymal fat tissue and the cell count per section (n = 3 per group). Scale bar: 100 µm. (**G**) Representative photographs of HE-stained sections of perirenal fat tissue and the cell count per section (n = 3 per group). Scale bar: 100 µm. All data represent means ± SEM.

**Figure 6:**
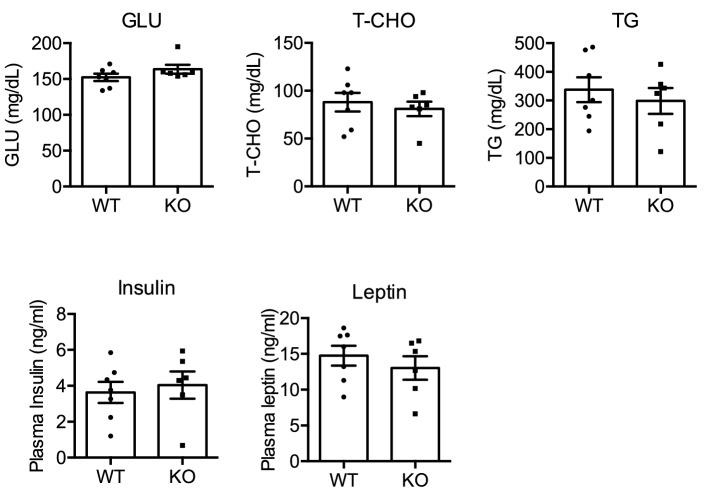
Blood biochemistry of male WT and NMU KO rats fed a high fat diet. Serum levels of GLU, T-CHO, and TGs were measured by an automatic biochemistry analyzer. Plasma levels of insulin and leptin were measured by ELISAs. All data represent means ± SEM (n = 6).

Next, we examined the effects of NMU deficiency on obesity and appetite after high fat chow feeding. Both WT and KO rats were fed a high fat diet for 11 weeks from weaning to 16 weeks of age. However, no difference was detected between WT and NMU KO rats at any measured age (Fig. 5A). Furthermore, the daily food consumption of NMU KO rats was not different from that of WT littermates (Fig. 5B). Further detailed analysis of adiposity showed no significant difference in the liver (Fig. 5C,E) or visceral fat (Fig. 5D,F,G). Blood tests showed that plasma levels of GLU, T-CHO, and TGs did not differ between WT and KO rats (Fig. 6). Circulating insulin and leptin levels were also not different between WT and NMU KO rats (Fig. 6). These results indicated no difference in susceptibility to obesity between WT and NMU KO rats, even when fed a high fat diet.

### Effects of NMU deficiency on feeding regulatory hormone expression in the hypothalamus

**Figure 7:**
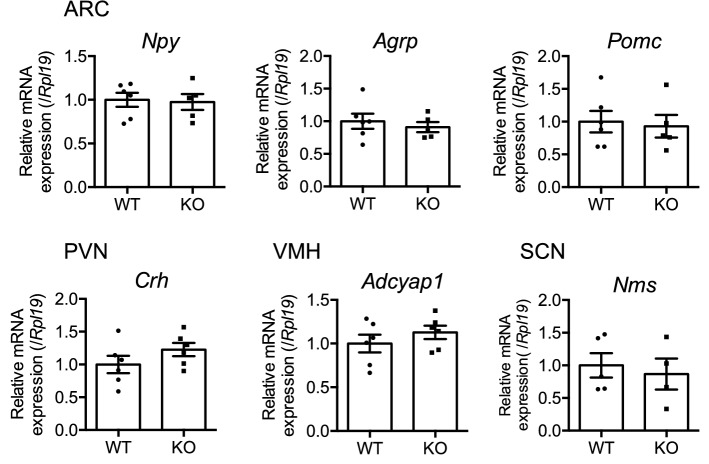
Expression levels of hypothalamic feeding regulatory hormones in male WT and NMU KO rats. Each brain region was collected by LMD. RT-qPCR analyses of *Npy*, *Agrp*, and *Pomc* were performed using ARC cDNA samples. RT-qPCR analysis of *Crh* was performed using PVN cDNA samples. RT-qPCR analysis of *Adcyap1* was performed using VMH cDNA samples. RT-qPCR analysis of *Nms* was performed using SCN cDNA samples. All data represent means ± SEM (n = 6). See also Supplementary Figure S2 .See also Supplementary Table S1 for primers.

NMU KO rats were unaffected in terms of the regulation of food intake and metabolic balance, and therefore other mechanisms may have compensated for NMU deficiency in NMU KO rats. Therefore, RT-qPCR analysis of LMD samples collected from specific regions (Supplementary Figure S2) was performed to examine the expression levels of several hypothalamic feeding regulatory hormone mRNAs. However, the expression levels of orexigenic factors *Npy* and *Agrp*, and anorectic factor *Pom*c in the ARC region and the anorectic factor *Crh* in the PVN region were not different between NMU KO and WT rats (Fig. 7). In addition, since neuromedin S (NMS) is known as an endogenous agonist of NMUR2^33^, NMS may have compensated for NMU deficiency in NMU KO rats. Nevertheless, the expression levels of *Nms* mRNA determined by RT-qPCR using LMD samples did not differ between WT and NMU KO rats (Fig. 7).

### *Nmu- and Nmur2 *mRNA-expressing region in the rat brain

**Figure 8:**
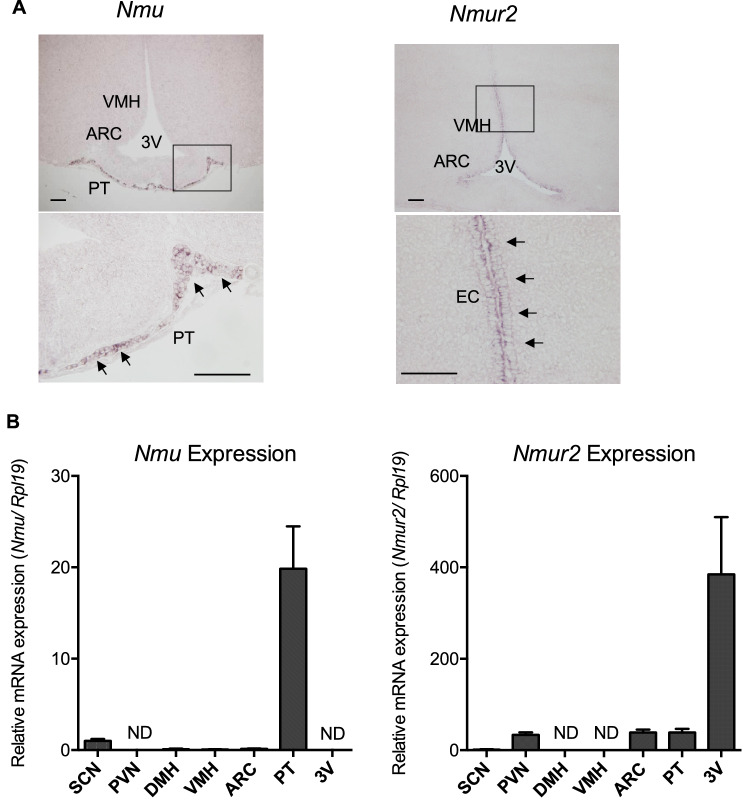
Expression of *Nmu and Nmur2* in WT rats. (**A**) Localization of *Nmu* and *Nmur2* mRNA expression determined by ISH in the rat brain. Representative microphotographs of ISH staining with DIG-labeled cRNA probes in a frontal section of the WT rat brain. Positive signals are indicated by arrows in magnified images (lower). Scale bar: 100 µm. VMH, ventromedial hypothalamic nucleus; ARC, arcuate nucleus; PT, pars tuberalis; 3 V, third ventricle. EC, ependymal cell layer of 3 V. (**B**) RT- qPCR analysis of *Nmu* and *Nmur2* expression in the SCN, PVN, DMH, VMH, ARC, PT, and EC using LMD samples. All data represent means ± SEM (WT n = 6 KO n = 5). ND indicates no signal detection. See also Supplementary Figure S2.

NMU KO rats showed no changes in metabolic regulation, raising questions about the mRNA expression regions of *Nmu* and *Nmur2* in the rat hypothalamus. To clarify the expressing region, we performed ISH and RT-qPCR of *Nmu* and *Nmur2*. As a result, intense staining of *Nmu* mRNA was observed in the PT of WT rats (Fig. 8A). However, no signal was observed in other regions in which *Nmu* expression has been reported to be weak using highly sensitive radioisotope-labeled probes, such as suprachiasmatic nucleus (SCN), dorsomedial hypothalamic nucleus (DMH), and arcuate nucleus (ARC)^25,34,35^. Because non-RI detection sensitivity using the DIG-labeled cRNA probe was not very high, we next examined expression by RT-qPCR using LMD samples. In addition to high levels in PT, *Nmu* expression was detected at low levels in SCN, which were close to the detection limit in DMH, ventromedial hypothalamic nucleus (VMH) and ARC, and was not detected in PVN (Fig. 8B).

In the rat hypothalamus, *Nmur2* expression was mainly observed in the ependymal layer of the third ventricle (3 V) (Fig. 8A). Although no signals were observed in ISH, RT-qPCR analysis of LMD samples revealed slight expression in PVN, ARC, and PT, and much lower levels in SCN, which appeared to be close to the detection limit in SCN, and no expression was detected in DMH or VMH (Fig. 8B).

## Discussion

By generating NMU KO rats using the CRISPR/Cas9 system and rGONAD method, we demonstrated that NMU deficiency in rats does not cause hyperphagia, hypertrophy, or fat deposition compared with WT littermates. Additionally, the combination of in situ hybridization and RT-qPCR confirmed that expression of *Nmu* and its receptor, *Nmur2*, was not detected or very low, which was probably close to the detection limit, in rat hypothalamic nuclei involved in regulation of feeding and energy metabolism. Our data indicate that rats NMU is not critically required for regulation of feeding and energy metabolism, and is likely not a central regulator of food intake.

Although NMU is a multifunctional peptide, it is believed that the main function of NMU is to regulate energy balance, which is strongly supported by the phenotypes of genetically modified mice. NMU-deficient mice develop significant obesity caused by hyperphagia and low energy metabolism, including reduced locomotor activity, energy expenditure, and decreased body temperature^20^. Hanada et al*.* also demonstrated that NMU KO mice have decreased *Crh* mRNA expression in the PVN, suggesting that CRH neurons are the main target of the NMU signaling pathway. CRH in the PVN is well known to reduce appetite and increase energy expenditure by stimulating sympathetic nerve activity. Therefore, obesity caused by NMU deficiency in mice may be attributed in part to the reduction of this anorectic peptide CRH^20^. Additionally, Graham et al. reported high expression of *Nmu* in the DMH of obese ob/ob mice, and the *Nmu* mRNA level in the DMH of WT mice is elevated by fasting^35^. These reports support the hypothesis that in mouse NMU is involved in the hypothalamic feeding regulation network.

Our results in rats were, however, inconsistent with those in mice. The cause of this discrepancy may be due to species-specific differences in the expression regions of *Nmu* and *Nmur2*. In the mouse hypothalamus, *Nmu* is expressed in ARC, DMH, and VMH, and *Nmur2* is expressed in PVN, DMH, VMH, and ARC^35^. These distributions of expression suggest that a high probability that NMU regulates feeding behavior through the neuronal circuits constituted by these hypothalamic regions. However, our study indicated that *Nmu* and *Nmur2* mRNAs in these hypothalamic nuclei, which are related to regulation of food intake and energy balance, were hardly detectable. Our data agree with those of Graham et al. (2003) who reported important differences in *Nmu* and *Nmur2* expression sites between the mouse and rat hypothalamus^34,35^. They demonstrated that *Nmu* mRNA is abundantly expressed in the mouse DMH and VMH, but only weakly in the rat DMH. *Nmu* is expressed in the ARC and SCN in both mice and rats, although its expression is much lower in rats. Thus, it appears reasonable that NMU deficiency in rats impairs neither body weight nor food intake. Although the peptide sequence of NMU is highly conserved among species, the species-specific expression pattern may allow NMU to have distinct functions across species. Nevertheless, we still need to take several points into consideration, functional protein levels could be different from the mRNA expression levels, especially for receptors. In this study, we detected *Nmur2* mRNA expression at very weak level in the PVN and ARC. Further understanding of NMUR2 in the PVN and ARC requires detailed analysis of NMUR2 protein expression, such as immunohistochemistry. Furthermore, feeding regulatory neuronal network is constructed not only by the hypothalamus but also by the cerebral cortex, midbrain, pons and medulla oblongata. Distribution studies in the rat brain revealed a wide distribution of NMU and NMU receptor other than in hypothalamus. Detailed ISH analysis showed *Nmu* expression in the nucleus of the solitary tract (NTS), which is in the control of feeding via the gastric and intestinal signals, and NMU expression in the NTS decreased in the obese Zucker fatty rats^28^. The NMUR2 expression in the dorsal raphe nucleus (DRN) was also demonstrated by immunostaining, suggesting that DRN is the site of NMU action^36^. The DRN is involved in the regulation of energy expenditure by brainstem GABA neurons^37^. The possibility of NMU in these nuclei for the regulation of energy intake/expenditure needs to be addressed in future studies.

Consistent with other studies in rats, we confirmed that *Nmu* expression was restricted in the rat brain and abundant in the PT of the pituitary^24,28,30,35,38,39^. Interestingly, in mice, *Nmu* expression in the PT has not been reported. Although NMU in the rat PT appears abundant, its physiological function remains to be clarified. We previously revealed the circadian rhythm of *Nmu* expression in the rat PT and proposed that NMU may be involved in the feeding rhythm such as rats eating food mostly at night^30^. In contrast to our previous expectation, this study showed that NMU deficiency did not impair the circadian pattern of feeding. Thus, in rats, our data suggest that endogenous NMU has no physiological function in the regulation of food consumption or the circadian feeding pattern.

Regarding the central NMU receptor^40^, *Nmur2* expression in the ependymal cell layer of 3 V was abundant in rats, which has also been reported in mice. The ependymal cell layer of the 3 V is composed with tanycytes that project onto several hypothalamic nuclei^41,42^. Although it must be elucidated in future studies, this indicates that endogenous NMU may have a common function in rats and mice. This study highlighted not only the similarities but also the considerable differences in the expression patterns for NMU and NMU2R in the hypothalamus of rats and mice.

In humans, there are limited data obtained on *Nmu* and *Nmur2* expression in the brain. The RT-qPCR analysis for *Nmu* expression in human CNS showed that in addition to the pituitary, several cortical areas of the brain such as the cingulate gyrus and medial frontal gyrus also express moderate levels of *Nmu.* Low to moderate levels on *Nmu* expression were also observed in many other brain regions, including the hypothalamus^43^. *Nmur2* expression in the substantia nigra and thalamus were observed by northern blotting^25^ and that in the hippocampus, hypothalamus, and cerebral cortexthat were detected by RT-qPCR^40^. At present, the lack of detail information , such as an absolutely clear picture image, on human sites of expression makes it impossible to identify which animal model may be more appropriate to infer physiological and behavioral responses to NMU in humans^8^. Together with our observations that NMU KO rats lacked significant differences in phenotypes regarding growth and appetite, we propose that it is necessary to carefully examine whether NMU is a critical factor involved in hypothalamic regulation of energy homeostasis and whether an NMU agonist could be an efficacious anti-obesity agent.

Several limitations of our study need to be considered, and we cannot rule out the possibility of compensatory responses for NMU deficiency in NMU KO rats. Further investigations of other rat models, such as conditional NMU knockout rats and injection of neutralizing antibody against NMU into wild type rats, may be necessary to fully support our conclusions.

In conclusion, we have demonstrated that NMU deficiency in rats does not impair their body weight, amount of food intake, or energy balance. Taken together with the expression pattern of *Nmu* and *Nmur2* in the rat hypothalamus, we propose that NMU in rats is not essential for regulation of food intake or energy balance.

## Methods

### Animals

F344 and WKY/NCrl rats were obtained from Charles River Laboratories Japan, Inc. (Kanagawa, Japan). The rats were maintained in a 12-h light/dark cycle (light switched on at 08:00) at room temperature (23 ± 2 °C) with food and water provided ad libitum. Animal experiments were approved by the Animal Care and Use Committee at Okayama University (permission number: #OKU-2017523, OKU-2017592, OKU-2020016, OKU-2020144, OKU-2020536, OKU-2020840) and Shigei Medical Research Institute (permission number: #17007, #20005), and were performed in accordance with the Guidelines for Animal Experimentation at Okayama University and Shigei Medical Research Institute. During the study and the care, housing and use of animals were strictly performed in accordance with the appropriate guidelines and regulations. All efforts were made to minimize animal suffering and to reduce the number of animals used in the experiments. Reporting of animal data in this study followed the recommendations set out in the ARRIVE guidelines.

### Generation of NMU KO rats

NMU KO rats were generated by the rGONAD method as described previously^31,32^. Pre-estrous F344 female rats were mated with male rats overnight and then copulation plugs were visually inspected the following morning and used for electroporation experiments. Electroporation experiments were operated on anesthetized female rats (Intraperitoneal injection of an anesthetic agent: mix of Midazolam, Vetorphale and Domitor). Single-stranded DNA containing three stop codons (5ʹ-TAGCTAGCTAGAATTCCCGG-3ʹ), Cas9 guide RNA against the first exon of *Nmu* (5ʹ-CGAGCAGCTAATCGCCGCCCAGG-3ʹ) (Fig. 1A), and Cas9 protein were injected into the oviductal lumen of pregnant female rats (E0.75) with the Alt-RTM CRISPR- Cas9 system (Integrated DNA Technologies, Coralville, IA) and subsequently electroporated by NEPA21 (Neppa Gene. Co., Ltd. Chiba, Japan). Guide RNA was designed using CHOPCHOP (http://chopchop.cbu.uib.no/). Gene alterations were verified by PCR and then DNA sequencing. Genomic DNA was isolated from an ear piece. Sequencing was performed using the PCR products. The PCR products were purified and directly sequenced and analyzed on an Applied Biosystems 3500 DNA sequencer (Thermo Fisher Scientific) using the BigDye Terminator v3.1 Cycle Sequencing Kit (Thermo Fisher Scientific). The selected founder generation rat was crossed with a wildtype rat to generate F1 generation *Nmu* + /- rats. WT and NMU KO rats were generated by crossing *Nmu* + /- rats. Genotyping was performed by PCR with genomic DNA isolated from an ear piece. PCR amplification was performed using EmeraldAmp MAX PCR Master Mix (Takara Bio Inc., Shiga, Japan) in accordance with the manufacturer’s instructions. Oligonucleotide-specific primers for WT and KO alleles were *Nmu-*Fw (5′-GATTTAAAAGTTGGTGGCGCG-3′) and *Nmu*-Rv (5′-GACAGGAGAGGAGATGCAGTT-3′). Amplicon sizes were confirmed by 2% agarose gel electrophoresis (product sizes: WT allele, 222 bp; KO allele; 242 bp) (Fig. 1D).

### Body weight measurement

Rats were maintained as two animals per cage after weaning. Body weight was measured weekly from 4 to 24 weeks of age during normal chow feeding (MF, Oriental Yeast Co., Ltd, Shiga, Japan) and from 4 to 15 weeks of age during high fat chow feeding (D12451, Research diet, Inc., NJ, USA).

### Food and water intake

Rats were maintained in an individual cage from 8 weeks of age and with food and water provided ad libitum. The amounts of food and water intake were measured weekly from 9 to 24 weeks of age to calculate average daily amounts. To analyze circadian patterns of food and water intake, we assessed light and dark phases of food intake for 4 days continuous and calculated the average amounts.

### Home cage locomotor activity and body temperature

Locomotor activity and body temperature were measured using the E-mitter telemetry system (Starr Life Sciences Corp., PA, USA). Rats were subcutaneously implanted with G2 E-transmitters under isoflurane anesthesia. Rats were individually maintained in plastic home cages [276 × 445 × 204 (H) mm; CLEA Japan, Inc., Tokyo, Japan] with food and water provided ad libitum. Data were collected for 4 continuous days from 5 days after the operation to calculate average activity volumes and mean body temperature.

### Preparation of an anti-NMU monoclonal antibody

We produced a rat monoclonal antibody against rNMU as described previously^44^. Briefly, a synthetic peptide of 19 amino acid residues with the sequence YKVNEYQGPVAPSGGFFLF-Cys, which corresponds to a part of the C-terminal side of rat mature NMU, was conjugated to keyhole limpet hemocyanin and an antigen emulsion was injected into WKY/NCrl rats anesthetized with sevoflurane. The treated rats were euthanized 17 days after the injection, and lymphocytes were fused with SP2/0-Ag14 myeloma cells. After the cell fusion, culture supernatants were screened to confirm positive clones by a solid-phase enzyme-linked immunosorbent assay (ELISA).

The specificity for the newly raised antibody was confirmed by western blot analysis to recognize synthetic rat NMU peptide (Cat. No. 350285, Abbiotec, Escondido, CA). Synthetic rat NMU peptide (3 ng) was subjected to 16% SDS-PAGE and transferred onto PVDF blotting membranes. The membranes were blocked with 5% dry skim milk in Tris-buffered saline containing 0.1% Tween-20 for 1 h. Immunoblotting was performed with the anti-NMU antibody, followed by a horseradish peroxidase-conjugated secondary antibody (1:5000 dilution). Immune complexes were visualized by enhanced chemiluminescence (Thermo Fisher scientific) using a ImageQuant 800 (Cytiva). Single bands corresponded to the predicted sizes (Supplementary Figure S1). Immunoreactivities had completely disappeared with a synthetic peptide-absorbed antibody.

### Immunohistochemistry

Immunohistochemical detection of NMU was carried out by the avidin–biotin complex (ABC) method. Male WT and NMU KO rats were sacrificed by decapitation under isoflurane anesthesia and brains were quickly removed. Brain tissues fixed with 4% paraformaldehyde were dehydrated in an ascending ethanol series, immersed in xylene, embedded in paraffin (Palaplast, Sakura Finetek USA, Inc., CA, USA), and sectioned at a thickness of 10 µm. The sections were dewaxed, incubated for 2 h with 10 mM sodium citrate (pH 6.0) for antigen retrieval, treated with 0.5% sodium metaperiodate to block endogenous peroxidases for 15 min, and then incubated with TNBS (1% normal horse serum and 0.4% Triton X-100 in PBS) for 1 h. After washing with PBS, the sections were incubated overnight in a humid chamber with the anti-NMU antibody diluted at 1:10 in TNBS. The ABC method was carried out with a staining kit (VECTASTAIN Elite ABC Kit Peroxidase; VECTOR Laboratories, CA, USA). The reactions were visualized with 0.02% 3ʹ3-diaminobenzidine tetrachloride in 0.006% H_2_O_2_ in 50 mM Tris-HCl, pH 7.6. Stained sections were viewed under a light microscope (BX60; Olympus, Tokyo, Japan) and photographed with a digital camera (DP70; Olympus).

### Hematoxylin and eosin staining

Male WT and NMU KO rats were sacrificed by decapitation under isoflurane anesthesia. Liver and adipose tissues were fixed in 4% paraformaldehyde, dehydrated in an ascending ethanol series, immersed in xylene, embedded in paraffin, and sectioned at a thickness of 10 µm. The sections were dewaxed, washed with DW and stained with hematoxylin and eosin (3 min for each staining). Stained sections were washed in running tap water, dehydrated with alcohol series and with xylene, and then mounted for light microscopic observation and imaging with a digital camera. For adipose tissue analysis, three regions were randomly selected in each section and photographed, and the cell number was counted to calculate the average cell number.

### *In situ* hybridization (ISH)

Male F344 rats were sacrificed by decapitation under isoflurane anesthesia and brains were quickly removed. ISH was performed on frozen 8-μm-thick frontal sections as described previously^30,45^. Digoxigenin-labeled antisense and sense rat *Nmu* cRNA probes (GenBank Accession No. NM_022239; positions 231–625) and rat *Nmur2* cRNA probes (GenBank Accession No. NM_022275; positions 473–1270) were synthesized using a labeling kit (Roche Diagnostics GmbH, Mannheim, Germany) with SP6 or T7 RNA polymerase.

### Laser microdissection (LMD) and preparation of cDNA samples

Male F344 rats were sacrificed by decapitation under isoflurane anesthesia and brains were quickly removed. Samples of the rat suprachiasmatic nucleus (SCN), PVN, dorsomedial hypothalamic nucleus (DMH), VMH, arcuate nucleus (ARC), PT, and ependymal cell layer of the third ventricle (3 V) were collected from serial frontal sections of rat brains (30 µm thick) using an LMD system (LMD 7000; Leica Microsystems, Wetzlar, Germany) as described previously^30,46^. Briefly, the sections were fixed with ice-cold acetone for 2 min, rehydrated by an ethanol series, and stained with 0.1% toluidine blue. Representative micrographic images of dissections are shown in Supplementary Figure S3. Total RNA from the samples was extracted using an RNeasy Micro Kit (QIAGEN GmbH, Hilden, Germany). cDNA was synthesized from the total RNA using Prime Script RT Master Mix (Takara Bio).

### RT-quantitative PCR (qPCR)

RT-qPCR was performed using the LightCycler 96 System (Roche Diagnostics) with SYBR Premix Ex Taq (Takara Bio). The primers used for RT-qPCR are shown in Supplementary Table S1. Rat *Rpl19* expression was evaluated as an internal control. The amplicon size and specificity were confirmed by a melting curve analysis and 2% agarose gel electrophoresis.

### Blood tests

Plasma glucose (GLU), total cholesterol (T-CHO), and triglycerides (TGs) were analyzed using a Hitachi 7180 automatic analyzer (Hitachi High-Technologies Corporation, Japan) with Wako commercial kits (FUJIFILM Wako Shibayagi Corp., Gunma, Japan). The analysis was performed by Oriental Yeast Co., Ltd. Insulin levels were measured using an Rebis Insulin-rat T ELISA kit (FUJIFILM Wako Shibayagi). Leptin levels were measured using a Leptin ELISA kit (Morinaga Institute of Biological Science, Yokohama, Japan).

### Statistical analysis

Data are presented as means ± SEM. Comparisons between two groups were performed by Student’s t test. All statistical analyses were performed using GraphPad Prism 6 software (GraphPad Software, La Jolla, CA). *P* < 0.05 was considered statistically significant.

## Supplementary Information

Supplementary Information.

## Data Availability

All data generated and analyzed during this study are included in this published article and its Supplementary Information.
