# Supplementary material for: Neuromedin U-deficient rats do not lose body weight or food intake
Source: Sci Rep. 2022 Oct 27;12:17472. doi: 10.1038/s41598-022-21764-6 (PMC9614009; doi:10.1038/s41598-022-21764-6)
Supplement: Supplementary file 1 — Supplementary Information. [file 41598_2022_21764_MOESM1_ESM.pdf]

Supplementary Table S1. Primer Sequences used for RT-qPCR

**Primers**	**Sequences (5’→3’)**	**GenBank accession No.**
Nmu, Forward	CGT TCC TCA ACT GCA TGA GA	NM_022239
Nmu, Reverse	CCA TTG CGT GGC CTA AAT AA	
Nmur2, Forward	GCG AAC AAA GTG GCT GTG AA	NM_022275
Nmur2, Reverse	GTC CAG CAG ATG GCA AAC AC	
Npy, Forward	GCC AGA TAC TAC TCC GCT CTG	NM_012614
Npy, Reverse	GTC TCA GGG CTG GAT CTC TTG	
Pomc, Forward	CCA TAG ACG TGT GGA GCT GG	NM_139326
Pomc, Reverse	AGG GCT GTT CAT CTC CGT TG	
Crh, Forward	ACC TGC CAA GGG AGG AGA A	NM_031019
Crh, Reverse	CCC TGC AAG GCA GAC AGG	
Rpl19, Forward	ACC AAC GAA ATC GCC AAT GC	NM_031103
Rpl19, Reverse	CAA GGT GTT CTT CCG GCA TC	
Adcyap1, Forward	AAC AGC GTC TCC TGT TCA CC	NM_016989
Adcyap1, Reverse	CCG TCC TGA TCG TAA GCC TC	
Agrp, Forward	TCC CAG AGT TCT CAG GTC TAA GT	NM_033650
Agrp, Reverse	CGC GGT TCT GTG GAT CTA GC	
Nms, Forward	TGG TCC TCC TGA TGG TTT GG	NM_001012233
Nms, Reverse	TTC CAA GCT CGG GAG TAG TG	

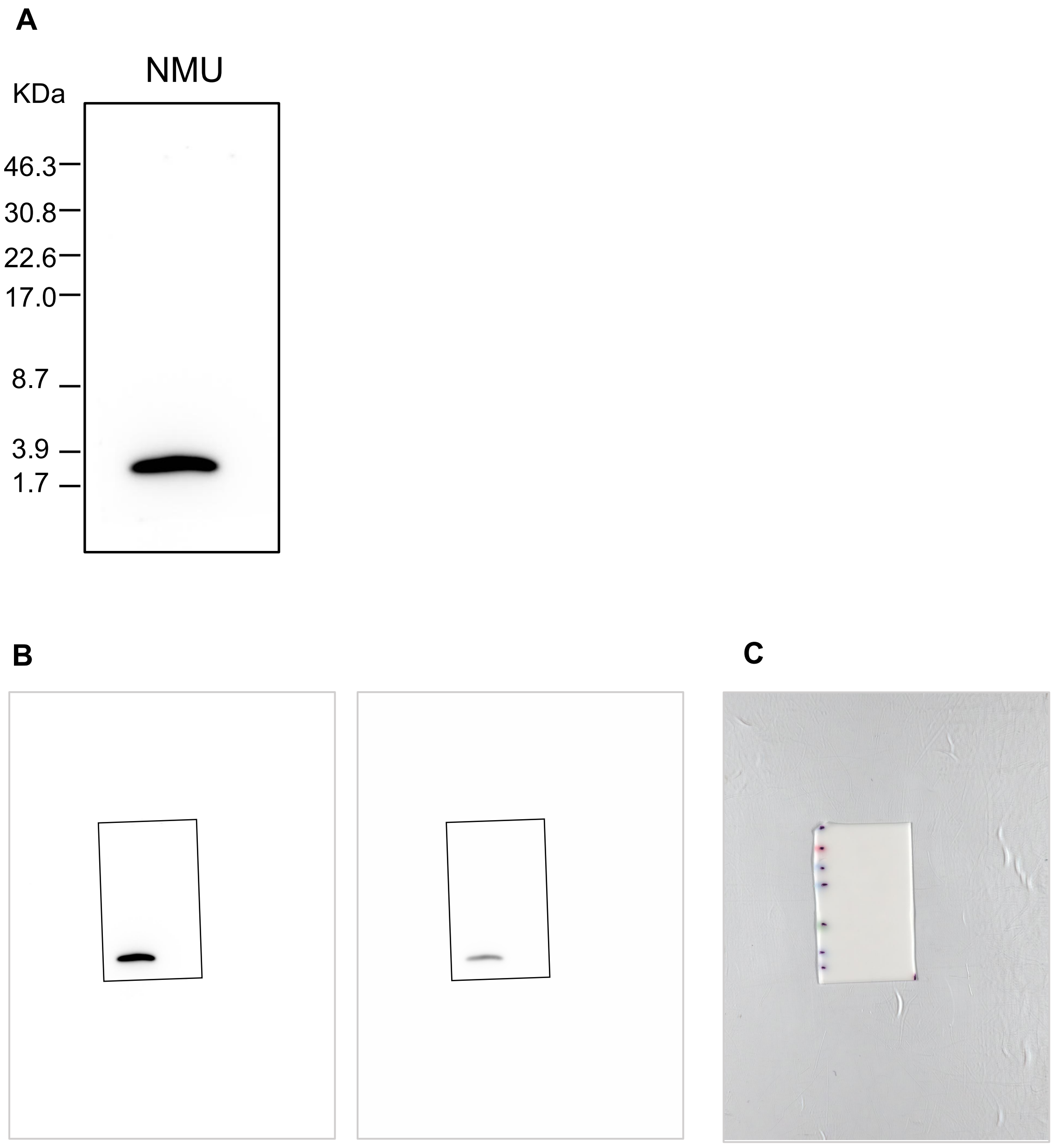

Supplementary Figure S1. Characterization of an antibody that specifically recognizes NMU. A. Autoluminogram of western blot detection of immunoreactivity for synthetic rat NMU peptide (2643.03 g/mol, 3 ng per lane). B. Original immunoblot images with different exposures. A black line indicates the border of the membrane. C. Original image of the membrane with molecular size marker (DynaMarker Protein MultiColor Stable, Low Range).

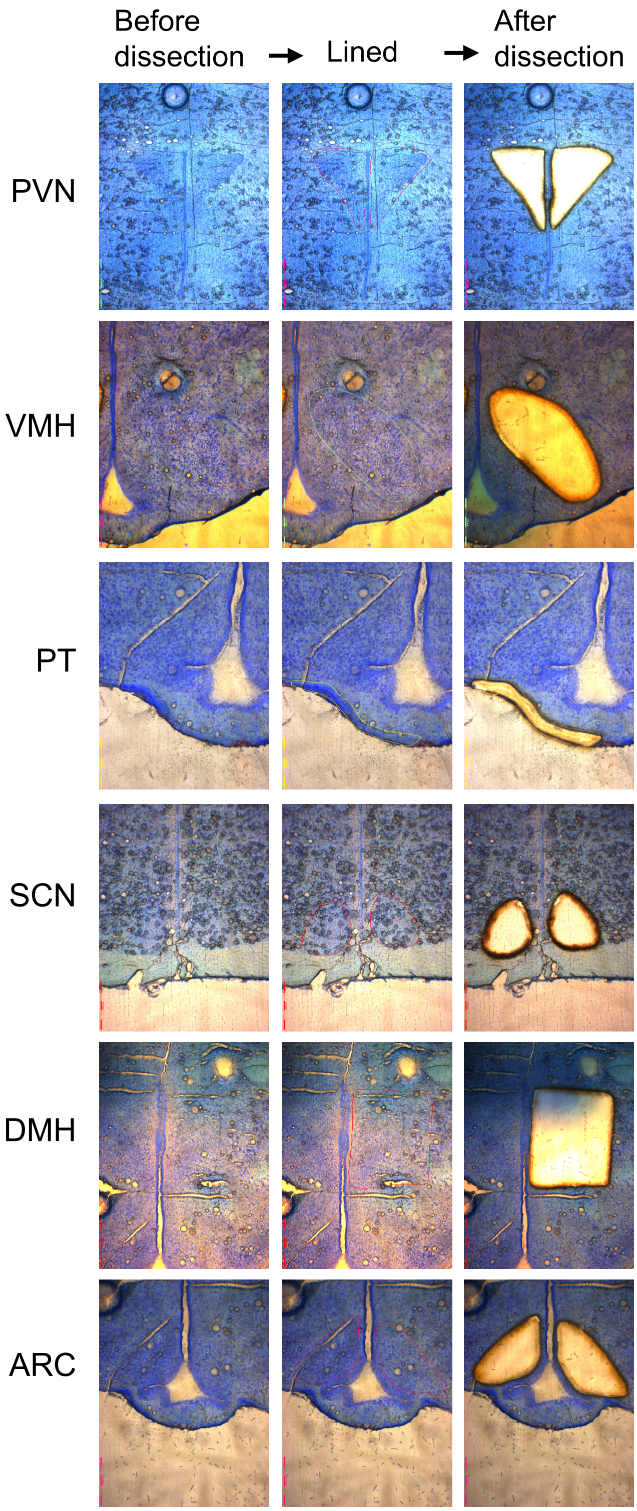

Supplementary Figure S2. Dissection of the PVN, VMH, PT, SCN, DMH, and ARC by LMD. Toluidine blue-stained 30-µm-thick frontal sections before dissection (left), lined (middle), and after dissection (right) are shown.

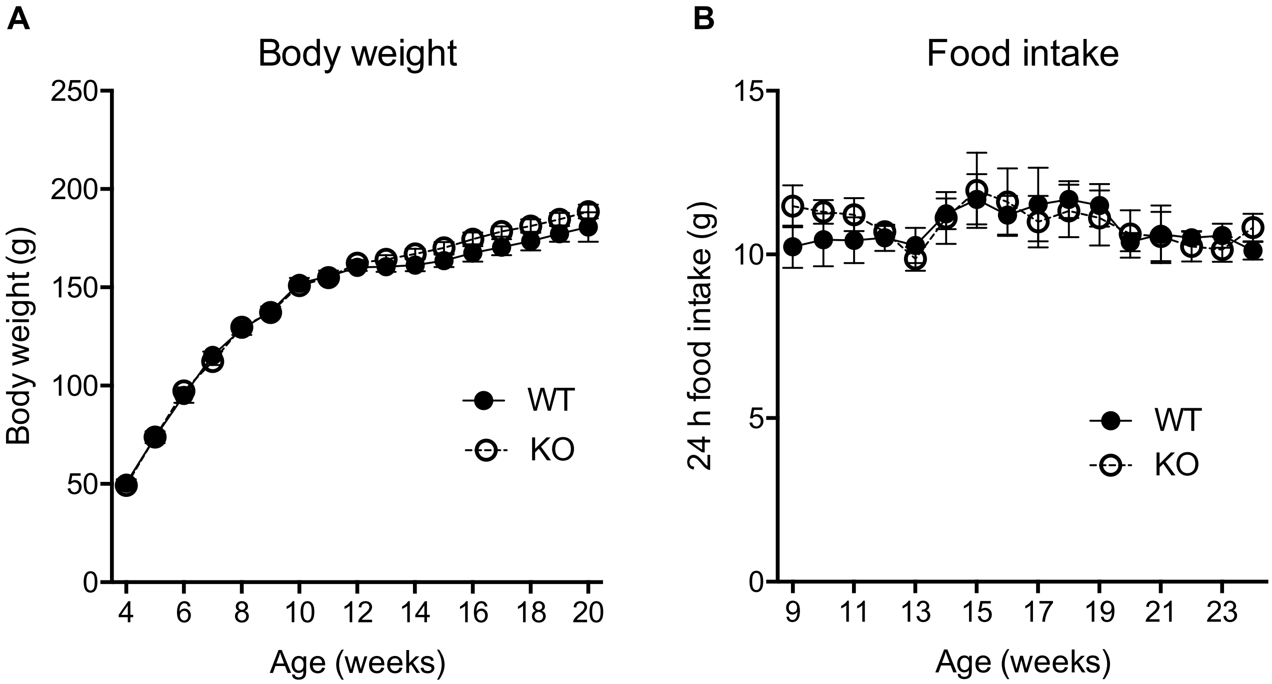

Supplementary Figure S3. Body weight and food consumption of female WT rats and KO rats. (A) Growth curve of WT rats (n=11) and KO rats (n=12) from 4 to 20 weeks of age. (B) Food intake of free-feeding rats from 9 to 24 weeks of age (WT n=11, KO n=12).
